# Predicting Retinopathy of Prematurity Risk Using Plasma Levels of Insulin-like Growth Factor 1 (IGF1), Tumor Necrosis Factor-Alpha (TNF-Alpha), and Neonatal Parameters

**DOI:** 10.3390/clinpract14040122

**Published:** 2024-08-01

**Authors:** Daniela Mariana Cioboata, Oana Cristina Costescu, Aniko Maria Manea, Florina Marinela Doandes, Mihaela Zaharie, Zoran Laurentiu Popa, Sergiu Costescu, Florina Stoica, Marioara Boia

**Affiliations:** 1 Department of Neonatology, “Victor Babes” University of Medicine and Pharmacy Timisoara, Eftimie Murgu Square 2, 300041 Timisoara, Romania; cioboata.daniela@umft.ro (D.M.C.); costescu.oana@umft.ro (O.C.C.); manea.aniko@umft.ro (A.M.M.); doandes.florina@umft.ro (F.M.D.); mihaela.zaharie@umft.ro (M.Z.); boia.marioara@umft.ro (M.B.); 2 Doctoral School Department, “Victor Babes” University of Medicine and Pharmacy Timisoara, Eftimie Murgu Square 2, 300041 Timisoara, Romania; 3 Department of Obstetrics and Gynecology, “Victor Babes” University of Medicine and Pharmacy Timisoara, 300041 Timisoara, Romania; sergiu.costescu@umft.ro; 4 Department of Ophthalmology, Emergency Municipal Clinical Hospital, 300172 Timisoara, Romania; florinastoica@umft.ro

**Keywords:** Retinopathy of Prematurity, respiratory distress syndrome, newborn, Insulin-like Growth Factor, Tumor Necrosis Factor, biomarkers

## Abstract

Background/Objectives: Retinopathy of Prematurity (ROP) remains a leading cause of vision impairment in premature infants, especially those with Respiratory Distress Syndrome (RDS) necessitating respiratory support. This study aimed to identify correlations between plasma levels of Insulin-like Growth Factor 1 (IGF1) and Tumor Necrosis Factor-alpha (TNF-alpha), and the risk of developing ROP. Additionally, it explored the association of ROP severity grades with plasma levels of glucose, lactate dehydrogenase (LDH), creatin phosphokinase (CPK), and other biomarkers, aiming to uncover predictive markers for ROP risk and severity in this population. Methods: This prospective study included premature neonates admitted with RDS requiring respiratory support, conducted over 18 months at the Neonatal Intensive Care Unit of the Louis Turcanu Emergency Clinical Hospital for Children, Timisoara. Plasma levels of IGF1 and TNF-alpha were measured on days 1 and 14 post-birth, alongside the initial assessment of glucose, LDH, and CPK levels. Results: Significant correlations were observed between lower gestational age and elevated LDH levels on day 7–10 (rho = −0.341, *p* = 0.0123) and between TNF-alpha levels at 2 weeks and ROP severity (rho = 0.512, *p* = 0.0004). Elevated IGF1 levels were protective against ROP, with Beta coefficients of 0.37 (*p* = 0.0032) for the first collection and 0.32 (*p* = 0.0028) for the second, suggesting their potential as biomarkers for ROP risk assessment. Higher levels of TNF-alpha at 2 weeks were associated with an increased risk of ROP (Beta = −0.45, *p* = 0.0014), whereas higher IGF1 levels offered protective effects against ROP, with Beta coefficients of 0.37 (*p* = 0.0032) for the first collection and 0.32 (*p* = 0.0028) for the second. Elevated LDH levels on day 7–10 post-birth were linked to an increased risk of ROP (Beta = 0.29, *p* = 0.0214). Conclusions: These findings highlight the potential of IGF1 and TNF-alpha as predictive biomarkers for ROP, offering avenues for early intervention and improved management strategies in this high-risk group.

## 1. Introduction

Retinopathy of Prematurity (ROP) remains a significant cause of childhood blindness worldwide, affecting the most vulnerable population of premature neonates [1,2]. The pathophysiology of ROP is complex, involving the interplay of genetic, metabolic, and environmental factors, with oxygen therapy and respiratory support being pivotal [3,4]. Recent advancements in neonatal care have improved survival rates of premature infants; however, this has concurrently increased the incidence of ROP, highlighting the necessity for accurate predictive markers for early identification and intervention. Insulin-like Growth Factor 1 (IGF1) and Tumor Necrosis Factor-alpha (TNF-alpha) have emerged as useful biomarkers due to their roles in angiogenesis and inflammatory pathways, respectively, which are crucial in the pathogenesis of ROP [5,6].

Extensive research has demonstrated the involvement of IGF1 in retinal vascular development and maturation, while TNF-alpha, a pro-inflammatory cytokine, has been implicated in the exacerbation of retinal neovascularization, suggesting its potential role as a biomarker for ROP risk [7,8]. The dynamic balance between these markers reflects not only the disease’s multifactorial nature but also the potential interventional points where therapeutic strategies could be targeted [9,10]. Therefore, understanding the correlation between these plasma levels and ROP development could offer a groundbreaking approach to managing this condition.

Moreover, other biological markers such as Lactate Dehydrogenase (LDH), Creatine Phosphokinase (CPK), and glucose levels have been studied in the context of ROP, indicating a broader metabolic disturbance in premature neonates at risk [11,12,13]. These markers, involved in energy metabolism and cellular injury, may provide additional insights into the systemic condition of these infants, further refining risk assessment models.

Real-world data underscore the urgency and the potential impact of this research. Globally, an estimated 15 million infants are born preterm each year, with a significant proportion developing ROP [14]. In regions with advanced neonatal care, the survival of extremely low-birth-weight infants (<1000 g) has dramatically increased, along with the prevalence of ROP [15,16]. This scenario presents a growing challenge to healthcare systems and calls for innovative approaches to predict, prevent, and manage this potentially blinding condition effectively.

Therefore, this study aims to establish a correlation between plasma levels of IGF1 and TNF-alpha in predicting the risk of developing ROP in premature neonates with respiratory distress syndrome treated with various modalities of respiratory support. It also explores the association between different ROP severity grades and plasma levels of glucose, LDH, CPK, and other biological markers. Through this approach, we seek to identify potential predictive biomarkers that can guide clinical interventions, improving the prognosis and management of ROP in this high-risk population.

## 2. Materials and Methods

### 2.1. Study Design

This research was designed as a prospective study, conducted over the span of 18 months from January 2021 to June 2022. The study was carried out in the Neonatal Intensive Care Unit (NICU) of the Louis Turcanu Emergency Clinical Hospital for Children in Timisoara. The cohort consisted of premature neonates who were admitted on their first day of life with a diagnosis of RDS and required respiratory support.

This study was conducted in accordance with the ethical standards of the institutional research committee and with the 1964 Helsinki declaration and its later amendments of ethical standards. The study protocol was reviewed by the Ethical Committee for Scientific Research, “Victor Babes” University of Medicine and Pharmacy Timisoara and approved on 28 September 2018 with the approval number 31. Informed parental consent was obtained from all individual parents of the participants included in the study.

### 2.2. Inclusion and Exclusion Criteria

The study specifically targeted premature neonates with a gestational age (GA) of 32 weeks or less who required ventilatory support from the time of admission. Importantly, the inclusion criteria did not impose any restrictions regarding birth weight, acknowledging that the risk and severity of ROP are influenced by multiple factors, including but not limited to the infant’s weight at birth. This inclusive approach ensured that the study could comprehensively evaluate the correlations between plasma levels of IGF1, TNF-alpha, and other biological markers with the development and severity of ROP across a diverse premature neonatal population.

To maintain the study’s integrity and focus, several exclusion criteria were established, comprising the presence of congenital malformations of the heart, eyes, or central nervous system, which could independently affect the development and progression of ROP or interfere with the study’s outcomes. Additionally, infants with congenital infections such as syphilis, rubella, cytomegalovirus, or toxoplasmosis were excluded due to the known impacts these conditions can have on neonatal health and development, potentially confounding the study’s findings [17,18]. Genetic syndromes, infants who died before the initial ophthalmologic examination, premature neonates admitted to the Neonatal Intensive Care Unit (NICU) after the first day of life, cases where informed consent was not obtained from the parents or guardians, and cases where parental psychiatric conditions prevented the acquisition of informed consent were also criteria for exclusion.

### 2.3. Study Protocol and Definitions

#### 2.3.1. Blood Sample Collection and Analysis

The study protocol outlined the procedures for collecting and analyzing blood samples for IGF1 and TNF-alpha levels. Blood samples were drawn on the first day of life and again on day 14, utilizing a venous approach to collect 2 mL in a vacutainer without anticoagulant but with a gel separator. Following collection, the samples were immediately centrifuged and subsequently stored at −70 degrees Celsius within the hospital’s analytical laboratory until further processing. To ensure accuracy and reproducibility, all samples were analyzed in duplicate, adhering to stringent laboratory practices.

#### 2.3.2. Biomarker Quantification

For the quantification of IGF1 levels, the DRG IGF-1 600ELISA kit from DRG Instruments GmbH, Marburg, Germany, was employed. This kit is a manual enzyme immunoassay designed for the quantitative measurement of IGF1 in human serum, reflecting the latest advancements in biochemical analysis. Similarly, TNF-alpha levels were determined using the TNF-alpha ELISA kit, also from DRG Instruments GmbH, Germany. This kit represents an immunoenzymatic assay crafted for the in vitro quantitative measurement of TNF-alpha in serum, ensuring the study’s measurements adhered to the highest standards of precision and reliability.

#### 2.3.3. Neurodevelopmental and Ophthalmologic Assessments

Additionally, all premature neonates requiring respiratory support underwent transfontanellar ultrasonography to diagnose intraventricular hemorrhage and periventricular leukomalacia. The Philips PureWave CX50 ultrasound system (Koninklijke Philips NV, Eindhoven, The Netherlands) was the instrument of choice for these critical diagnostic evaluations. This sophisticated equipment facilitated the early detection and monitoring of these serious conditions, further augmenting the study’s comprehensive approach to understanding the multifaceted nature of ROP risk factors. This protocol not only emphasized the importance of biochemical markers but also integrated crucial neurodevelopmental assessments, painting a more complete picture of the health challenges faced by premature neonates.

In order to classify ROP, this study adhered to the guidelines set by the International Classification of Retinopathy of Prematurity [19]. This classification system delineates ROP into distinct stages based on the severity of the condition: Stage 1, marked by a visible demarcation line between vascularized and non-vascularized retina; Stage 2, characterized by the presence of a ridge replacing the demarcation line; Stage 3, which involves extraretinal fibrovascular proliferation or neovascularization; Stages 4A and 4B, indicating partial retinal detachment; and Stage 5, denoting total retinal detachment. Additionally, the classification acknowledges the presence of plus disease, a sign of disease severity marked by venous dilation and arterial tortuosity in the posterior retinal vessels. Aggressive Posterior ROP (AP-ROP), a rare, rapidly progressing and severe form of ROP [20], is highlighted for its posterior location, significant plus disease, and the ill-defined nature of the retinopathy, often leading to Stage 5 ROP if left untreated. This detailed classification system is pivotal for accurately assessing the progression of ROP and tailoring interventions accordingly.

In our study, the respiratory support administered to premature neonates included High-Flow Nasal Cannula (HFNC), Nasal Continuous Positive Airway Pressure (nCPAP), and Synchronized Intermittent Mandatory Ventilation (SIMV) or Synchronized Intermittent Positive Pressure Ventilation (SIPPV). These modalities were chosen based on the individual clinical needs of the neonates to ensure optimal respiratory assistance and closely monitored to adapt to the dynamic requirements of their respiratory conditions.

Patients were evaluated by an ophthalmologist using indirect ophthalmoscopy. The initial examination was conducted at 4 weeks post-birth or at 30–31 weeks postmenstrual age, with subsequent examinations scheduled based on the disease’s severity. To facilitate these examinations, mydriatic solutions (0.5% tropicamide or 2.5% phenylephrine) were administered. The findings from each examination were meticulously documented in the patient’s observation sheet, ensuring a comprehensive and detailed record of the progression or improvement of ROP. This rigorous evaluation protocol allowed for the timely identification of ROP stages and any signs of aggressive disease progression, enabling appropriate and potentially sight-saving interventions.

#### 2.3.4. Documentation and Data Management

Biochemical markers such as glucose, LDH, and CPK were collected on the first day of life upon admission to the NICU. The inclusion of these markers in the study protocol underscores the multifaceted approach to understanding ROP’s pathophysiology. By correlating these biochemical markers with ROP’s clinical severity, the study aims to unveil potential systemic associations that could further refine risk stratification and management strategies for this vulnerable population. This holistic approach to patient evaluation combines detailed ophthalmic assessments with systemic biochemical analysis, reflecting the complex interplay of factors contributing to ROP development and progression.

### 2.4. Statistical Analysis

Data management and analysis were conducted utilizing the statistical software SPSS version 26.0 (SPSS Inc., Chicago, IL, USA). Continuous variables are represented as mean ± standard deviation (SD), while categorical variables are expressed in terms of frequencies and percentages. The Student’s *t*-test was used for comparing two means between the normally distributed data, and the Mann–Whitney u-test was used for non-Gaussian data, respectively. The Chi-square test was utilized for the categorical variables. A Pearson correlation was calculated to test associations between continuous variables, and a regression analysis was performed to determine risk factors for ROP. A *p*-value threshold of less than 0.05 was set for statistical significance. All results were double-checked to ensure accuracy and reliability.

## 3. Results

### 3.1. ROP vs. Non-ROP

In Table 1, we examined the background characteristics of neonates categorized into two groups: those with any stage of ROP (*n* = 47) and those without ROP (*n* = 48). The distribution of gender across groups was not statistically significant, with 48.9% males in the Any ROP group compared to 72.9% in the No ROP group (*p* = 0.134). A notable difference was observed in the gestational age of neonates, where the Any ROP group had a mean gestational age of 31.9 weeks (SD = 1.6), which was significantly lower than the 30.6 weeks (SD = 2.1) observed in the No ROP group, with a *p*-value of 0.0010. 

The birth weight of neonates did not show a statistically significant difference between the two groups, with the Any ROP group having a mean birth weight of 1527 g (SD = 275) compared to 1494 g (SD = 228) in the No ROP group (*p* = 0.5255). APGAR scores at 1 min post-birth indicated a statistically significant difference, with the Any ROP group having a lower mean score (6.2, SD = 2.0) compared to the No ROP group (7.1, SD = 1.4), with a *p*-value of 0.0126. However, the APGAR scores at 5 min, while still lower in the Any ROP group (6.9, SD = 1.8) compared to the No ROP group (7.5, SD = 1.6), did not reach statistical significance (*p* = 0.0891). 

Monitored pregnancies were significantly less prevalent in the Any ROP group (48.9%) compared to the No ROP group (72.9%), with a *p*-value of 0.0165. This association may indicate a link between less monitored pregnancies and increased ROP risk. On the other hand, the mode of birth, as indicated by cesarean birth rates, did not significantly differ between groups (55.3% in Any ROP vs. 60.4% in No ROP; *p* = 0.6320).

Lactate dehydrogenase levels were measured at two different time intervals: day 1–3 and day 7–10 post-birth. Initially, LDH levels did not show a statistically significant difference between the groups (869 ± 552 U/L in the Any ROP group vs. 740 ± 614 U/L in the No ROP group; *p* = 0.2846). However, by day 7–10, LDH levels in the Any ROP group were significantly higher (599 ± 268 U/L) compared to those in the No ROP group (435 ± 253 U/L), with a *p*-value of 0.0028. 

The study also investigated glucose levels, finding that neonates with ROP had lower mean glucose levels (3.1 ± 1.5 mmol/L) compared to those without ROP (3.8 ± 1.9 mmol/L), with the difference reaching statistical significance (*p* = 0.0495). Also, CPK levels, another marker investigated, did not significantly differ between the groups (282 ± 180 U/L in Any ROP vs. 259 ± 166 U/L in No ROP; *p* = 0.5188).

A noteworthy finding of the study was the significantly higher levels of TNF-alpha, a pro-inflammatory cytokine, in the Any ROP group both on the first day of life (24.9 ± 18.8 pg/mL vs. 14.2 ± 12.3 pg/mL in No ROP; *p* = 0.0014) and at 2 weeks of life (38.2 ± 45.3 pg/mL vs. 16.9 ± 24.0 pg/mL in No ROP; *p* = 0.0051). Lastly, the analysis of IGF1 levels revealed significantly lower IGF1 levels in the Any ROP group both at the first collection (61.4 ± 35.6 ng/mL) and at the second collection (57.9 ± 32.8 ng/mL) compared to the No ROP group (91.6 ± 44.6 ng/mL and 90.1 ± 48.2 ng/mL, respectively), with *p*-values of 0.0004 and 0.0003 (Table 2).

The utilization of High-Flow Nasal Cannula (HFNC) therapy showed no significant difference between the two groups, with 17.0% in the Any ROP group and 16.7% in the No ROP group (*p*-value = 0.9631). Similarly, the application of Nasal Continuous Positive Airway Pressure (nCPAP) was relatively balanced between the groups, with 46.8% in the Any ROP group compared to 52.1% in the No ROP group (*p*-value = 0.6071).

Synchronized Intermittent Mandatory Ventilation (SIMV) or Synchronized Intermittent Positive Pressure Ventilation (SIPPV) usage was observed in 36.2% of neonates with ROP versus 31.3% without ROP, with a *p*-value of 0.6119. The duration of oxygen therapy, a critical factor in ROP management, showed no significant difference between the groups, with neonates in the Any ROP group receiving an average of 7.8 days (SD = 4.1) of therapy compared to 7.4 days (SD = 3.6) in the No ROP group (*p* = 0.6143). 

White matter injury, a severe complication, was observed in 38.3% of neonates with ROP and 33.3% of those without, resulting in a non-significant *p*-value of 0.6137. The prevalence of Intraventricular Hemorrhage (IVH) was also analyzed, with 12.8% in the Any ROP group and 10.4% in the No ROP group, yielding a *p*-value of 0.7204. Moreover, a significant finding of this study was the use of laser treatment, exclusively administered to neonates in the Any ROP group (17.0%), with none in the No ROP group undergoing this intervention, resulting in a *p*-value of 0.0028 (Table 3). 

### 3.2. Analysis by ROP Grades

The distribution of gender among the groups revealed a statistically significant difference (*p* = 0.0156), with a higher percentage of males in the non-ROP group (72.9%) and ROP Aggressive Posterior group (80.0%), compared to the lower percentages observed in the ROP Grade I and II (48.5%) and ROP Grade III (33.3%) groups. Gestational age showed a significant variation across the groups (*p* = 0.0227), with the ROP Grade III group having the lowest mean gestational age (29.0 weeks, SD = 1.7), followed by the ROP Aggressive Posterior group (30.6 weeks, SD = 1.5) and the non-ROP group (30.6 weeks, SD = 1.4). The ROP Grade I and II group had a slightly higher mean gestational age (31.0 weeks, SD = 1.2). 

Birth weight also varied significantly across the groups (*p* = 0.0089), with the lowest mean birth weight observed in the ROP Grade III group (1274 g, SD = 215). The ROP Aggressive Posterior and non-ROP groups had similar mean birth weights (1556 g, SD = 204, and 1494 g, SD = 231, respectively).

The APGAR scores at 1 min (*p* = 0.0294) and 5 min (*p* = 0.0367) post-birth showed statistical significance across the groups. The ROP Grade III and ROP Grade I and II groups had lower APGAR scores compared to the non-ROP group. Monitored pregnancies were less frequent in the groups with ROP, with a statistically significant difference (*p* = 0.0471). The rate of Cesarean births, although not statistically significant (*p* = 0.0553), varied across the groups, with the highest percentage observed in the ROP Grade I and II group (66.7%), as described in Table 4.

Lactate dehydrogenase levels were evaluated at two intervals: day 1–3 and day 7–10. An exceptional elevation was noted in the LDH levels during days 1–3 for the ROP Grade III group (1659.6 ± 394 U/L), significantly higher than the other groups, with a *p*-value of 0.0065. This pronounced increase suggests that severe ROP might be associated with higher levels of cellular distress or injury early post-birth. Conversely, LDH levels during days 7–10, while still elevated in the ROP Grade III group, showed a more widespread significance across the groups (*p* = 0.0320), indicating ongoing cellular stress beyond the immediate postnatal period. Glucose levels across the groups revealed a statistically significant difference (*p* = 0.0462), with the lowest levels observed in the ROP Grade III group (3.0 ± 1.4 mmol/L). 

Creatine phosphokinase levels demonstrated a marked increase in the ROP Grade III group (450.4 ± 229 U/L), significantly differing from the other groups (*p* = 0.0184). The study also investigated levels of TNF-alpha. Notably, TNF-alpha levels on the first day of life were highest in the ROP Aggressive Posterior group (69.7 ± 22.5 pg/mL), suggesting an early inflammatory response in neonates who develop severe forms of ROP (*p* = 0.0012). Insulin-like Growth Factor 1 (IGF1) levels were assessed at two points, revealing significantly lower levels in neonates with ROP compared to those without, both initially and subsequently (*p*-values of 0.0138 and 0.0044, respectively), as presented in Table 5. 

The utilization of HFNC and nCPAP did not exhibit a statistically significant difference across the groups, with *p*-values of 0.7584 and 0.2104, respectively. However, the application of SIMV/SIPPV showed a significant disparity (*p* = 0.0017), particularly highlighting an elevated usage in neonates diagnosed with ROP Aggressive Posterior (80.0%) and ROP Grade III (66.7%) compared to those with ROP Grade I and II (21.2%) and without ROP (31.3%). The duration of oxygen therapy revealed a significant difference across the groups (*p* = 0.0088), with neonates in the ROP Grade III group receiving the longest duration of therapy (12.1 ± 3.1 days), implying that prolonged exposure to oxygen therapy might be associated with higher severity levels of ROP. 

Analysis of white matter injury and intraventricular hemorrhage showed no significant associations with ROP severity, with *p*-values of 0.3146 and 0.8893, respectively. A striking result was observed in the administration of laser treatment, where a significant difference was noted (*p* < 0.0001). All neonates with ROP Aggressive Posterior received laser treatment, and a substantial proportion of those with ROP Grade III were treated as well (33.3%), whereas none in the ROP Grade I and II and non-ROP groups received this intervention, as described in Table 6. 

### 3.3. Risk Factors

The analysis revealed a significant positive correlation between gestational age and birth weight (rho = 0.651, *p* = 0.0004), indicating that higher gestational ages are typically associated with higher birth weights. A strong positive correlation was also observed between APGAR scores at 1 min and 5 min post-birth (rho = 0.779, *p* < 0.0001), suggesting that neonates’ condition tends to remain consistent in the immediate post-birth period. 

Conversely, a negative correlation was identified between gestational age and LDH levels on day 7–10 (rho = −0.341, *p* = 0.0123), as well as between gestational age and TNF-alpha levels at 2 weeks (rho = −0.288, *p* = 0.0214). Interestingly, a positive correlation emerged between birth weight and both IGF1 measurements (first collection: rho = 0.512, *p* = 0.0012; second collection: rho = 0.489, *p* = 0.0023), suggesting that higher birth weights are associated with higher levels of IGF1. 

**Figure 1:**
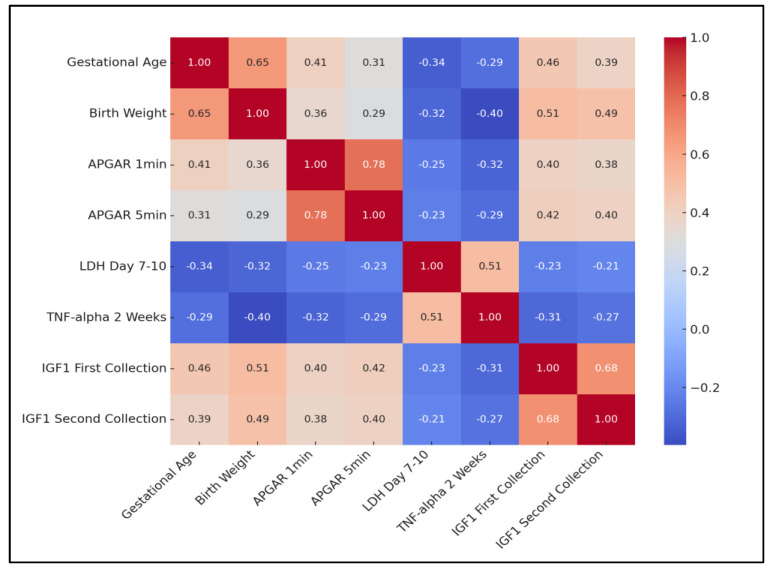
Correlation heatmap; LDH—Lactate Dehydrogenase; TNF—Tumor Necrosis Factor; IGF—Insulin-like Growth Factor; APGAR—Appearance, Pulse, Grimace, Activity, Respiration.

Moreover, TNF-alpha levels at 2 weeks showed a significant positive correlation with LDH day 7–10 (rho = 0.512, *p* = 0.0004), indicating that higher inflammatory responses are associated with greater cellular stress or damage. Finally, the strong positive correlation between IGF1 measurements at two different time points (rho = 0.678, *p* < 0.0001) indicates that IGF1 levels tend to remain consistent over time in neonates, as presented in Table 7 and Figure 1.

Gestational age emerged as a significant negative predictor for ROP, with a beta coefficient of −0.34 (*p* = 0.0123). This indicates that for each week increase in gestational age, the risk of ROP decreases, underlining the critical role of prematurity in ROP development. The confidence interval ranged from −0.536 to −0.144, reinforcing the robustness of this finding. Birth weight showed a positive association with decreased ROP risk, evidenced by a beta coefficient of 0.21 (*p* = 0.0427). This suggests that higher birth weights are protective against ROP, a finding supported by the confidence interval ranging from 0.0532 to 0.3668.

APGAR scores at 1 min and 5 min post-birth were also significant predictors, with beta coefficients of 0.15 (*p* = 0.0189) and 0.12 (*p* = 0.0345), respectively. These positive coefficients imply that higher APGAR scores, indicative of better neonatal condition at birth, are associated with a lower risk of ROP. Lactate dehydrogenase (LDH) levels on day 7–10 post-birth were positively correlated with an increased risk of ROP, demonstrated by a beta coefficient of 0.29 (*p* = 0.0214). This highlights the potential role of cellular injury or stress, as measured by LDH, in the pathogenesis of ROP. 

**Figure 2:**
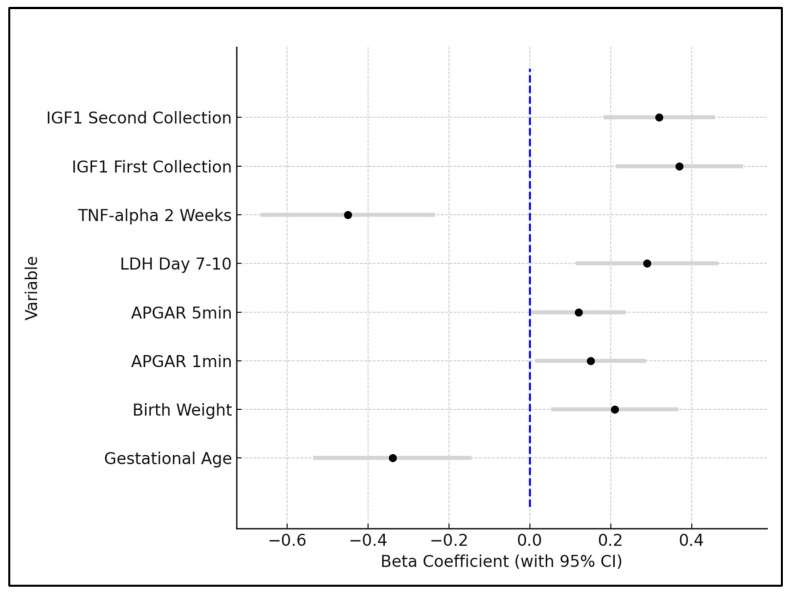
Forest plot analysis of risk factors for ROP development; LDH—Lactate Dehydrogenase; TNF—Tumor Necrosis Factor; IGF—Insulin-like Growth Factor; APGAR—Appearance, Pulse, Grimace, Activity, Respiration.

TNF-alpha levels at 2 weeks showed a significant negative relationship with ROP development, with a beta coefficient of −0.45 (*p* = 0.0014), suggesting that higher levels of this inflammatory marker are linked to an increased risk of ROP. Insulin-like Growth Factor 1 (IGF1) levels, both at the first and second collection, were found to be significant protective factors against ROP, with beta coefficients of 0.37 (*p* = 0.0032) and 0.32 (*p* = 0.0028), respectively. These findings indicate that higher levels of IGF1 are associated with a reduced risk of developing ROP, as seen in Table 8 and Figure 2. 

The sensitivity and specificity analysis of TNF-alpha and IGF1 as predictive biomarkers for ROP in premature neonates revealed that both markers had an AUC of 0.616, indicating a good predictive ability. The optimal threshold for TNF-alpha was identified at 24.9 pg/mL and for IGF1 at 31.1 ng/mL. While both markers demonstrated a high sensitivity of 88.2%, suggesting they are effective in identifying neonates with ROP, their specificity was only 63.7%, indicating a considerably lower ability in correctly identifying neonates without ROP. 

## 4. Discussion

The current study brought to light critical insights into the laboratory parameters associated with varying degrees of ROP severity. Through our analysis, it became evident that specific biochemical markers play a significant role in the progression and risk assessment of ROP. Notably, the study’s findings on lactate dehydrogenase levels, glucose concentrations, TNF-alpha, and IGF1 levels offer a nuanced understanding of the biological underpinnings that may influence ROP development. LDH levels, particularly on day 7–10 post-birth, showcased a notable increase in neonates with ROP compared to those without, highlighting a potential marker of cellular stress or damage in the context of ROP. This increase was most pronounced in the group with ROP Grade III, suggesting a correlation between elevated LDH levels and more severe forms of ROP. The role of LDH as an indicator of tissue breakdown or hypoxia aligns with the pathophysiology of ROP, where ischemic insult can precipitate retinal neovascularization [21,22].

Furthermore, the study’s findings on glucose levels reinforced the complexity of metabolic regulation in preterm infants and its potential impact on ROP development. Neonates with ROP exhibited lower glucose levels than their counterparts without ROP, indicating that hypoglycemia might be associated with an increased risk or severity of ROP. This relationship between glucose levels and ROP underscores the importance of glucose monitoring and management in preterm infants to potentially mitigate ROP risk.

The investigation into TNF-alpha levels provided compelling evidence of the role of inflammation in ROP. Significantly higher levels of TNF-alpha were observed in the ROP groups, especially on the first day of life and at 2 weeks, underscoring the inflammatory nature of ROP pathogenesis. This pro-inflammatory cytokine’s elevated levels in neonates with more severe ROP point to inflammation as a critical factor in disease progression, suggesting that anti-inflammatory strategies might hold therapeutic potential in managing ROP. Moreover, the analysis of IGF1 showed that lower levels of IGF1 were consistently found in neonates with ROP, highlighting its protective role against the disease, not only emphasizing the importance of IGF1 in vascular development and overall neonatal growth but also suggesting that increased IGF1 levels could be a potential strategy in preventing ROP or reducing its severity [23].

The association between IGF1/TNF-α and ROP is hypothesized to stem from their roles in inflammation and vascular regulation. TNF-α, a pro-inflammatory cytokine, may contribute to the vascular abnormalities seen in ROP by promoting inflammation and neovascularization, critical factors in the pathogenesis of ROP. Conversely, IGF1, known for its role in normal vascular development, may be protective by supporting vascular stability and integrity. Lower levels of IGF1 and elevated levels of TNF-α could disrupt normal retinal vascular development, thus predisposing neonates to ROP. Both IGF1 and TNF-α have been implicated in various complications associated with prematurity, such as bronchopulmonary dysplasia and necrotizing enterocolitis, primarily through their roles in inflammation and growth regulation. Our study specifically focused on the correlation of these biomarkers with ROP development. However, while collecting data, we accounted for potential confounders, including other neonatal outcomes and clinical interventions, to isolate the specific impact of IGF1 and TNF-α levels on ROP risk, ensuring a more accurate analysis of their roles in this specific condition.

Cakir’s et al. exploration of the relationship between hyperglycemia, insulin insensitivity, low IGF1 levels, and the development and severity of ROP in extremely preterm infants offers compelling evidence that supports our findings on the predictive value of IGF1 levels [23]. Their observation that the highest mean plasma glucose tertile correlated with increased ROP prevalence (34 of 39 neonates) and severity (71% with ROP stage 3 or higher) resonates with our study’s indication that lower IGF1 levels are associated with increased ROP risk. Moreover, their finding that recombinant human IGF1 (rh-IGF1) treatment reduced neovascularization and improved retinal revascularization underscores the therapeutic potential of IGF1, aligning with our conclusion that IGF1 levels play a crucial role in ROP development. Also, another study, focusing on a new method to identify newborns at risk of ROP based on IGF1 levels and the presence of sepsis, further validates our approach by demonstrating a 100% negative predictive value for ROP screening [24]. Their protocol, which effectively reduced unnecessary screenings by 79.1% without missing any ROP cases, provides a practical framework that complements our study’s emphasis on IGF1 as a biomarker for ROP risk.

Similarly, another study [25] illustrated a clear association between low postnatal serum IGF1 levels and severe ROP in a racially diverse U.S. cohort, adding an essential dimension to our study’s results, being among first in the Romanian region, by confirming the relevance of this association across varied populations. Their observation that mean IGF1 levels were lowest in infants developing stage 3 ROP (17.0 ng/mL) compared to those with no ROP (20.0 ng/mL) during PMA weeks 28–33, even after adjusting for birth weight and gestational age, resonates with our findings of significantly lower IGF1 levels being predictive of ROP development. Conversely, another study by Jafari et al. [26] did not find a significant association between IGF-1 levels and ROP, despite noting differences in IGF1 levels based on birth weight categories. This discrepancy could hint at the complexity of ROP’s pathophysiology, suggesting that while IGF1 is a critical factor, its impact might be modulated by other variables.

The findings from our study, emphasizing the predictive role of IGF1 and TNF-alpha levels in ROP development, resonate with insights from other recent investigations. The study by Tan et al. [27] underscored the complexity of ROP pathogenesis by categorizing potential biomarkers, including cytokines and growth factors, which aligns with our observation of lower IGF1 levels being significantly associated with ROP. Specifically, our analysis showing a substantial decrease in IGF1 levels in ROP cases (61.4 ng/mL for any ROP vs. 91.6 ng/mL for no ROP) mirrors the trends noted in the literature where low serum IGF1 was consistently linked to severe ROP outcomes. Concurrently, Hellgren’s et al. [28] longitudinal analysis revealed a negative correlation between IL-6 levels and IGF1, especially notable between 5 and 8 weeks post-birth, complementing our findings on the role of TNF-alpha. This parallel between the elevated pro-inflammatory markers and lower IGF1 in ROP development not only corroborates our results but also suggests an intricate interplay between inflammation and growth regulation in ROP pathogenesis. Noteworthy is the observed increase in IL-6 and TNF-α levels 24 h post-birth in infants who later developed ROP, which, alongside our findings of a significant relationship between TNF-alpha levels and ROP severity, underscores the potential of integrating inflammatory markers with IGF1 profiling to enhance ROP risk assessment and management strategies.

The clinical relevance of this study’s findings lies in the potential for improved ROP risk stratification and early intervention strategies for preterm neonates by assessing the IGF1. Additionally, the significant correlation between TNF-alpha levels and ROP severity suggests that inflammation plays a key role in ROP pathogenesis, opening suggestions for therapeutic interventions aimed at reducing inflammation. Importantly, these findings advocate for the integration of biomarker monitoring into routine neonatal care for preterm infants, facilitating a more personalized approach to ROP management. This approach not only has the potential to improve patient outcomes but also to optimize resource allocation by focusing efforts on neonates at highest risk, ultimately enhancing the quality of care for this vulnerable population.

The identification of solid predictive biomarkers for ROP can significantly enhance early intervention strategies. By accurately predicting the risk of ROP before the onset of visible symptoms, healthcare providers can tailor preventative measures, adjust the intensity of monitoring, and potentially initiate early treatments such as optimizing oxygen therapy and other modifiable factors. This proactive approach could reduce the incidence and severity of ROP, improving visual outcomes and reducing long-term complications associated with this condition.

One notable limitation of this study lies in its single-center design, which may constrain the generalizability of the findings to broader neonatal populations with varying demographic and clinical characteristics. Additionally, the study’s relatively small sample size, especially when stratifying neonates by the severity of ROP, may limit the statistical power to detect subtle differences or interactions between biomarkers and ROP outcomes. While the study meticulously controlled for a range of biological markers, it did not encompass all potential confounding variables, such as maternal health factors, prenatal care quality, and postnatal interventions other than respiratory support, which might influence ROP development. The reliance on blood samples at only two time points also may not fully capture the dynamic nature of changes in IGF1 and TNF-alpha levels and their relationship with ROP progression over time. Further research, preferably multi-centered with larger cohorts and a broader scope of investigated variables, is required to validate these findings and explore additional predictive biomarkers for ROP. Moreover, future analyses will incorporate statistical methods appropriate for repeated measures to better account for the correlations between measurements taken at different time points on the same subjects.

## 5. Conclusions

This study underscores the importance of TNF-alpha and IGF1 as significant predictive biomarkers for ROP in premature infants with RDS. The findings suggest that higher gestational age and birth weight are protective against ROP, while elevated levels of LDH and lower levels of IGF1 increase the risk. These biomarkers offer a promising avenue for early intervention and targeted management strategies in the high-risk premature neonate population, potentially mitigating the adverse outcomes associated with ROP.

## Figures and Tables

**Table 1:** Background characteristics of neonates (any ROP vs. no ROP).

Variables	Any ROP (*n* = 47)	No ROP (*n* = 48)	*p*-Value
Gender, *n* (%)			0.134 *
Male	23 (48.9%)	35 (72.9%)	
Female	27 (51.1%)	13 (27.1%)	
Gestational age, weeks (mean ± SD)	31.9 ± 1.6	30.6 ± 2.1	0.0010 **
Birth weight, grams (mean ± SD)	1527 ± 275	1494 ± 228	0.5255 **
APGAR score 1 min, (mean ± SD)	6.2 ± 2.0	7.1 ± 1.4	0.0126 **
APGAR score 5 min, (mean ± SD)	6.9 ± 1.8	7.5 ± 1.6	0.0891 **
Monitored Pregnancy, *n* (%)	23 (48.9%)	35 (72.9%)	0.0165 **
Cesarean Birth, *n* (%)	26 (55.3%)	29 (60.4%)	0.6320 *

* Chi-square or Fisher’s exact test; ** Student’s *t*-test; SD—standard deviation; ROP—Retinopathy of Prematurity; APGAR—Appearance, Pulse, Grimace, Activity, Respiration.

**Table 2:** Comparison of laboratory data (any ROP vs. no ROP).

Variables (Mean ± SD)	Any ROP (*n* = 47)	No ROP (*n* = 48)	*p*-Value *
LDH Day 1–3 (255–600 U/L)	869 ± 552	740 ± 614	0.2846
LDH Day 7–10 (255–600 U/L)	599 ± 268	435 ± 253	0.0028
Glucose (3.88–6.38 mmol/L)	3.1 ± 1.5	3.8 ± 1.9	0.0495
CPK (24–228 U/L)	282 ± 180	259 ± 166	0.5188
TNF-alpha First Day of Life	24.9 ± 18.8	14.2 ± 12.3	0.0014
TNF-alpha 2 Weeks of Life	38.2 ± 45.3	16.9 ± 24.0	0.0051
IGF1 First Collection	61.4 ± 35.6	91.6 ± 44.6	0.0004
IGF1 Second Collection	57.9 ± 32.8	90.1 ± 48.2	0.0003

* Student’s *t*-test; SD—standard deviation; LDH—Lactate Dehydrogenase; CPK—Creatine Phosphokinase; TNF—Tumor Necrosis Factor; IGF—Insulin-like Growth Factor; ROP—Retinopathy of Prematurity.

**Table 3:** Comparison of outcomes and interventions among neonates with ROP vs. no ROP.

Variables	Any ROP (*n* = 47)	No ROP (*n* = 48)	*p*-Value
HFNC, *n* (%)	8 (17.0%)	8 (16.7%)	0.9631 *
nCPAP, *n* (%)	22 (46.8%)	25 (52.1%)	0.6071 *
SIMV/SIPPV, *n* (%)	17 (36.2%)	15 (31.3%)	0.6119 *
Oxygen Therapy (days), (mean ± SD)	7.8 ± 4.1	7.4 ± 3.6	0.6143 **
White Matter Injury, *n* (%)	18 (38.3%)	16 (33.3%)	0.6137 *
Intraventricular Hemorrhage, *n* (%)	6 (12.8%)	5 (10.4%)	0.7204 *
Laser Treatment, *n* (%)	8 (17.0%)	0 (0.0%)	0.0028 *

* Chi-square or Fisher’s exact test; ** Student’s *t*-test; SD—standard deviation; HFNC—High-Flow Nasal Cannula; ROP—Retinopathy of Prematurity; CPAP—Continuous Positive Airway Pressure; SIMV—Synchronized Intermittent Mechanical Ventilation; SIPPV—Synchronized Intermittent Positive Pressure Ventilation.

**Table 4:** Comparison of background characteristics between different degrees of ROP severity.

Variable	ROP Aggressive Posterior (*n* = 5)	ROP Grade I and II (*n* = 33)	ROP Grade III (*n* = 9)	Non-ROP (*n* = 48)	*p*-Value
Gender, *n* (%)					0.0156 *
Male	4 (80.0%)	16 (48.5%)	3 (33.3%)	35 (72.9%)	
Female	1 (20.0%)	17 (51.5%)	6 (66.7%	13 (27.1%)	
Gestational age, weeks (mean ± SD)	30.6 ± 1.5	31.0 ± 1.2	29.0 ± 1.7	30.6 ± 1.4	0.0227 **
Birth weight, grams (mean ± SD)	1556 ± 204	1591 ± 189	1274 ± 215	1494 ± 231	0.0089 **
APGAR score 1 min, (mean ± SD)	5.2 ± 1.8	6.3 ± 1.5	6.3 ± 1.6	7.1 ± 1.3	0.0294 **
APGAR score 5 min, (mean ± SD)	6.8 ± 1.7	7.0 ± 1.4	7.0 ± 1.5	7.5 ± 1.2	0.0367 **
Monitored Pregnancy, *n* (%)	3 (60.0%)	16 (48.5%)	4 (44.4%)	35 (72.9%)	0.0471 *
Cesarean Birth, *n* (%)	0 (0.0%)	22 (66.7%)	4 (44.4%)	29 (60.4%)	0.0553 *

* Chi-square or Fisher’s exact test; ** Student’s *t*-test; SD—standard deviation; ROP—Retinopathy of Prematurity; APGAR—Appearance, Pulse, Grimace, Activity, Respiration.

**Table 5:** Comparison of laboratory data between different degrees of ROP severity.

Variables, (Mean ± SD)	ROP Aggressive Posterior (*n* = 5)	ROP Grade I and II (*n* = 33)	ROP Grade III (*n* = 9)	Non-ROP (*n* = 48)	*p*-Value *
LDH Day 1–3 (255–600 U/L)	773.8 ± 341	625.8 ± 308	1659.6 ± 394	840.3 ± 322	0.0065
LDH Day 7–10 (255–600 U/L)	550.3 ± 256	442.5 ± 213	680.1 ± 266	485.1 ± 247	0.0320
Glucose (3.88–6.38 mmol/L)	3.3 ± 1.2	3.1 ± 1.0	3.0 ± 1.4	3.8 ± 1.1	0.0462
CPK (24–228 U/L)	194.8 ± 104	249.1 ± 156	450.4 ± 229	268.5 ± 168	0.0184
TNF-alpha First Day of Life	69.7 ± 22.5	16.9 ± 24.6	29.3 ± 20.8	14.2 ± 19.0	0.001
TNF-alpha 2 Weeks of Life	34.7 ± 46.1	23.3 ± 40.3	23.6 ± 44.8	16.9 ± 37.5	<0.0001
IGF1 First Collection	73.3 ± 40.5	62.1 ± 42.3	52.1 ± 49.1	91.6 ± 41.6	0.0138
IGF1 Second Collection	53.1 ± 36.4	58.3 ± 44.8	58.5 ± 50.8	90.1 ± 44.0	0.0044

* Student’s *t*-test; SD—standard deviation; LDH—Lactate Dehydrogenase; CPK—Creatine Phosphokinase; TNF—Tumor Necrosis Factor; IGF—Insulin-like Growth Factor; ROP—Retinopathy of Prematurity.

**Table 6:** Comparison of laboratory data between different degrees of ROP severity.

Variable	ROP Aggressive Posterior (*n* = 5)	ROP Grade I and II (*n* = 33)	ROP Grade III (*n* = 9)	Non-ROP (*n* = 48)	*p*-Value
HFNC, *n* (%)	0 (0.0%)	8 (24.2%)	0 (0.0%)	8 (16.7%)	0.7584 *
nCPAP, *n* (%)	1 (20.0%)	18 (54.6%)	3 (33.3%)	25 (52.1%)	0.2104 *
SIMV/SIPPV, *n* (%)	4 (80.0%)	7 (21.2%)	6 (66.7%)	15 (31.3%)	0.0017 *
Oxygen Therapy (days), (mean ± SD)	8.6 ± 2.5	6.5 ± 2.0	12.1 ± 3.1	7.4 ± 1.8	0.0088 **
White Matter Injury, *n* (%)	1 (20.0%)	14 (42.4%)	3 (33.3%)	16 (33.3%)	0.3146 *
Intraventricular Hemorrhage, *n* (%)	0 (0.0%)	5 (15.2%)	1 (11.1%)	5 (10.4%)	0.8893 *
Laser Treatment, *n* (%)	5 (100%)	0 (0.0%)	3 (33.3%)	0 (0.0%)	<0.0001 *

* Chi-square or Fisher’s exact test; ** Student’s *t*-test; SD—standard deviation; HFNC—High-Flow Nasal Cannula; ROP—Retinopathy of Prematurity; CPAP—Continuous Positive Airway Pressure; SIMV—Synchronized Intermittent Mechanical Ventilation; SIPPV—Synchronized Intermittent Positive Pressure Ventilation.

**Table 7:** Correlation matrix.

Variables (Rho, *p*-Value)	Gestational Age	Birth Weight	APGAR 1 Min	APGAR 5 Min	LDH Day 7–10	TNF-Alpha 2 Weeks	IGF1 First Collection	IGF1 Second Collection
Gestational Age	1	0.651, 0.0004	0.408, 0.0126	0.311, 0.0891	−0.341, 0.0123	−0.288, 0.0214	0.457, 0.0032	0.392, 0.0079
Birth Weight		1	0.359, 0.0257	0.294, 0.1053	−0.315, 0.2991	−0.398, 0.0028	0.512, 0.0012	0.489, 0.0023
APGAR 1 min			1	0.779, <0.0001	−0.249, 0.3217	−0.317, 0.0246	0.403, 0.0051	0.376, 0.0075
APGAR 5 min				1	−0.233, 0.3389	−0.289, 0.0345	0.419, 0.0042	0.395, 0.0064
LDH Day 7–10					1	0.512, 0.0004	−0.233, 0.0427	−0.208, 0.0541
TNF-alpha 2 Weeks						1	−0.309, 0.0189	−0.274, 0.0256
IGF1 First Collection							1	0.678, <0.0001
IGF1 Second Collection								1

LDH—Lactate Dehydrogenase; TNF—Tumor Necrosis Factor; IGF—Insulin-like Growth Factor; APGAR—Appearance, Pulse, Grimace, Activity, Respiration.

**Table 8:** Risk factor analysis for ROP development.

Variables	Beta Coefficient	Standard Error	*p*-Value	Lower CI	Upper CI
Gestational Age	−0.34	0.10	0.0123	−0.536	−0.144
Birth Weight	0.21	0.08	0.0427	0.0532	0.3668
APGAR 1 min	0.15	0.07	0.0189	0.0128	0.2872
APGAR 5 min	0.12	0.06	0.0345	0.0024	0.2376
LDH Day 7–10	0.29	0.09	0.0214	0.1136	0.4664
TNF-alpha 2 Weeks	−0.45	0.11	0.0014	−0.6656	−0.2344
IGF1 First Collection	0.37	0.08	0.0032	0.2132	0.5268
IGF1 Second Collection	0.32	0.07	0.0028	0.1828	0.4572

LDH—Lactate Dehydrogenase; TNF—Tumor Necrosis Factor; IGF—Insulin-like Growth Factor; CI—Confidence Interval; APGAR—Appearance, Pulse, Grimace, Activity, Respiration.

## Data Availability

The original contributions presented in the study are included in the article, further inquiries can be directed to the corresponding author.
